# Cross-cultural adaptation and validation of the Canadian Haemophilia Outcomes-Kids’ Life Assessment Tool (CHO-KLAT) in Côte d’Ivoire (the Ivory Coast)

**DOI:** 10.1186/s12955-020-01327-x

**Published:** 2020-03-18

**Authors:** Catherine Lambert, N’ Dogomo Meité, Ibrahima Sanogo, Sébastien Lobet, Cedric Hermans, Séverine Henrard, Victor Blanchette, Nancy L. Young

**Affiliations:** 1 Haemostasis and Thrombosis Unit, Division of Hematology, Cliniques universitaires Saint-Luc, Brussels, Belgium; 2 Division of Clinical Hematology, Centre Hospitalier Universitaire de Yopougon, Abidjan, Côte d’Ivoire; 3 Division of Physical Medicine and Rehabilitation, Cliniques universitaires Saint-Luc, Brussels, Belgium; 4 Université catholique de Louvain (UCLouvain), Secteur des Sciences de la Santé, Institut de Recherche Expérimentale et Clinique, Neuromusculoskeletal Lab, 1200 Brussels, Belgium; 5 Clinical Pharmacy Research Group (CLIP), Louvain Drug Research Institute (LDRI), Université catholique de Louvain (UCLouvain), Brussels, Belgium; 6 Institute of Health and Society (IRSS), Université catholique de Louvain (UCLouvain), Brussels, Belgium; 7 Department of Pediatrics, University of Toronto, Toronto, ON Canada; 8 Division of Hematology/Oncology, Hospital for Sick Children, Toronto, ON Canada; 9 School of Rural and Northern Health, Laurentian University, Sudbury, ON Canada

**Keywords:** Haemophilia, Health-related quality of life, CHO-KLAT, Developing country

## Abstract

**Introduction:**

Health-related quality of life evaluation is recognized as an important outcome in the assessment of boys with haemophilia. In fact, reliable health-related quality of life data are even more critical in developing countries to advocate for government agencies to develop national haemophilia care programmes. However, validated tools are not yet available in sub-Saharan African countries.

**Aims:**

The purpose of this study was to complete the cultural adaptation and validation of the Canadian Haemophilia Outcomes-Kids’ Life Assessment Tool version_2.0_ (CHO-KLAT_2.0_) in Côte d’Ivoire.

**Methods:**

The process included four steps: a linguistic adaptation, cognitive debriefing interviews with children and their parents, a validity assessment with the Pediatric Quality of Life Inventory (PedsQL) as a comparator, and a test-retest reliability assessment.

**Results:**

The initial Ivoirian version of the CHO-KLAT_2.0_ was developed through a linguistic adaptation performed in close collaboration with members of the local medical team and haemophilia community. Cognitive debriefings were completed with five boys and their parents, with the final Ivoirian version of the CHO-KLAT_2.0_ developed in September 2017. The validation process included 37 boys with haemophilia (mean age: 11.4 years; 34 with severe and three with moderate forms of haemophilia, all treated on demand) and their parents. Among the child-reported population (*n* = 20), we observed a mean CHO-KLAT_2.0_ score of 51.3 ± 9.2; there was a moderate correlation between the CHO-KLAT_2.0_ and PedsQL scores (*r* = 0.581; *p* = 0.007) and an inverse correlation of the CHO-KLAT_2.0_ and PedsQL scores with the global rating of the degree to which the boys were bothered by their haemophilia. The mean parent proxy CHO-KLAT_2.0_ score (*n* = 17) was 53.5 ± 9.8. Among the parents, we found no significant correlation between the Ivoirian CHO-KLAT_2.0_ and PedsQL scores or between the parent-reported scores and the parent global ratings of bother. The test-retest intraclass correlation coefficient was 0.879 (95% CI: 0.673; 0.954) for the child-reported questionnaires and 0.880 (95% CI: 0.694; 0.955) for the proxy-reported questionnaires.

**Conclusions:**

A cross-culturally adapted and validated version of the CHO-KLAT_2.0_ for Côte d’Ivoire is now available that enables baseline values to be obtained and intervention outcomes (namely, prophylaxis) to be measured in Ivoirian boys with haemophilia.

## Introduction

Haemophilia is a congenital X-linked recessive bleeding disorder resulting in low levels of either factor VIII (FVIII, haemophilia A [HA]) or factor IX (FIX, haemophilia B [HB]). The condition occurs in approximately 1 in 5000 (HA) and 1 in 20,000–30,000 (HB) live male births [1]. Depending on residual coagulation factor activity, people with haemophilia (PWH) experience various degrees of bleeding, primarily affecting the joints, muscles, and soft tissues [2]. Recurrent joint bleeds cause long-term complications including pain, arthropathy, and disability [3]. The major goal in treating PWH is to reduce the frequency of bleeds and, consequently, mortality and joint damage. Treatment is based on replacement of the missing clotting factor when the bleed occurs (on demand) or preventively with regular prophylaxis. Hence, haemophilia-related complications and treatment have a major impact on health-related quality of life (HRQoL) [4]. Consequently, HRQoL is considered a key clinical outcome in the assessment of PWH [5, 6]. Given that HRQoL is closely linked to people’s culture, HRQoL tools developed in one context may not reflect how people from another culture view their health, and assessment tools often require cross-cultural adaptation and validation [5–7]. The process of the cross-cultural translation and adaptation of HRQoL measures is well-documented in the literature [8, 9], including modified methods that enable limitations, such as small sample sizes of children with rare disorders, to be overcome [10].

The Canadian Haemophilia Outcomes-Kids’ Life Assessment Tool (CHO-KLAT) is a disease- specific, child-centric, HRQoL measure for boys with haemophilia (BWHs) that requires few resources [11]. There is only a version for children aged 4 to 18 years old that includes both children’s self-reported and parent-proxy-reported questionnaires. The CHO-KLAT contains 35 items, is scaled from 0 to 100 [11], and has been validated against the Pediatric Inventory of Quality of Life (PedsQL), a generic HRQoL assessment tool [12]. The CHO-KLAT is valid, sensitive to significant clinical developments, and capable of assessing the impact of haemophilia-related interventions for HRQoL in a target population [12]. The CHO-KLAT has strong psychometric properties, with proven test-retest reliability [12], content validity [11], and high child-parent concordance [12]. It was initially developed in North American English and was then updated to a version_2.0_ [13] and cross-culturally adapted and validated in several European countries [14], as well as China [15, 16] and Brazil [17, 18].

Only a few studies have fully evaluated HRQoL in developing countries, where most PWH are treated on demand, resulting in repeated bleeding episodes with potentially negative impacts on HRQoL. Accurate data on HRQoL are, however, critical for advocating for government agencies to develop national haemophilia care programmes. However, in sub-Saharan African countries, research on haemophilia is limited [19], and the CHO-KLAT_2.0_ has been translated only into South African languages [20]. No HRQoL measurements have yet been cross-culturally validated in the Ivoirian haemophilia population, but French versions do exist. In 2016, there was no access to prophylaxis in Côte d’Ivoire and clotting factor concentrates were supplied by humanitarian aid with a mean per capita FVIII and FIX use of 0.032 and 0.005, respectively [21].

Our study sought to complete a cultural and linguistic adaptation of the CHO-KLAT version_2.0_, assess its validity and reliability in Côte d’Ivoire and, in doing so, obtain baseline values and enable the measurement of intervention outcomes in BWHs.

## Participants and methods

### Participants

This study was conducted from January 2017 to January 2018 at the Hemophilia Treatment Center (HTC) of the Yopougon University Hospital in Abidjan, which is the only HTC in Côte d’Ivoire. During multidisciplinary visits, we screened all 43 BWHs aged 4–18 years old who were being followed at the Yopougon HTC and their parents.

The study was conducted as part of the World Federation of Hemophilia twinning programme with a partnership established between the Ivoirian HTC of Yopougon and the international HTC of the Cliniques universitaires Saint-Luc in Brussels, Belgium.

### Methods

The translation and validation process followed a modified cross-cultural translation approach [10], comprising cultural and linguistic adaptation, cognitive debriefing interviews, and validity testing (including of the test-retest reliability) of the Ivoirian CHO-KLAT_2.0_. To determine whether to use either a proxy- or self-reported assessment among BWHs who were 8 to 12 years of age, literacy skills were evaluated before the administration of the questionnaires; meanwhile, the questionnaires were completed by parents for children aged 4 to 8 years old.

#### Linguistic adaptation

The Ivoirian version of the CHO-KLAT_2.0_ was based on the French CHO-KLAT_2.0_, as the official language in Côte d’Ivoire is French. The questionnaire was reviewed by two Ivoirian haematologists from the HTC, two members of the National Members’ Organization, two adolescent BWHs, and three parents of BWHs. All provided comments and proposed modifications that were reviewed by the Canadian CHO-KLAT development team and a Belgian physician experienced in treating haemophilia who was fluent in both French and English and was involved in the twinning programme. A translation of the Ivoirian CHO-KLAT_2.0_ into English was performed by three native speakers of the target language who had never seen the questionnaire and had the following expertise. This step was carried out to ensure the meaning of the original questionnaire was preserved after the linguistic adaptation. Any discrepancies were resolved by the Canadian team and Belgian-Ivorian partners.

#### Cognitive debriefing interviews

Cognitive debriefing (using Jobe’s framework [22]) was conducted to ensure that the initial Ivoirian version of the CHO-KLAT_2.0_ was well understood by both children and parents in all linguistic, cultural, and clinical contexts. The following inclusion criteria for the debriefing interviews were applied: boys aged 4–17.9 who had haemophilia A or B, with or without inhibitors, were native French speakers, did not have significant cognitive impairment, were able to read French, and had a parent who was available for interviews. BWHs and parents participated separately in face-to-face interviews and completed the initial Ivoirian version of the CHO-KLAT_2.0_ alongside a research team member. Any suggestions for improvements made by the respondents were recorded, and participants were asked if they felt any items were missing.

#### Data collection

All data were collected on site by the same medical team. The demographics that were obtained for all participating BWHs included age, type and severity of haemophilia, familial or sporadic form of haemophilia, treatment regimen, inhibitor status, place of residence, and education. In addition, in the sample of BWHs aged 4–18 who were recruited for the validity testing, body mass index (BMI) [23], and a musculoskeletal assessment was performed by a trained physiotherapist using the Hemophilia Joint Health Score (HJHS_2.1_), which is an 11-item scoring tool for assessing joint impairment in 4–18-year-olds [24]. Demographic data that were obtained from parent proxy respondents included family relationship with the BWH, occupation and education. Participants were administered the CHO-KLAT_2.0_ and PedsQL at baseline (T1) during a clinical visit and then a second time (T2) using the Ivoirian version CHO-KLAT_2.0_ 1–2 weeks after the initial administration. Only clinically stable children without active bleeds during this period were included in the test-retest analysis.

#### Statistical analysis

The validity of the final version of the Ivoirian CHO-KLAT_2.0_ was assessed against the PedsQL, a 23-item self-reported generic measure of HRQoL [25], which was chosen as a comparator because it was used to validate the original CHO-KLAT in Canada [12] and because a modified version had already been widely administered in Côte d’Ivoire [26]. This step was intended to demonstrate whether the previously established correlation between the CHO-KLAT and PedsQL [12] was maintained with the Ivoirian version. The CHO-KLAT_2.0_ and PedsQL summary scores were calculated according to the questionnaire manuals. Both the CHO-KLAT_2.0_ and PedsQL are scaled 0 to 100, with a score of 100 indicating the best HRQoL. The strength of the correlations between the Ivoirian CHO-KLAT_2.0_ and the other instruments were calculated qualitatively using Pearson’s correlation coefficient (r).

Pearson’s correlation was computed to assess the relationship between the two HRQoL measures and a global rating of the degree to which BWHs were bothered by haemophilia. This rating of bother was assessed by responses ranging from “not at all” to “very much”. The internal consistency of the items of the Ivoirian CHO-KLAT_2.0_ was assessed using Cronbach’s alpha for the child self-reports and parent proxy reports. Notably, since the CHO-KLAT is a clinimetrically derived tool, high internal consistency was not expected.

The test-retest reliability between the T1 and T2 CHO-KLAT_2.0_ scores was assessed using an intraclass correlation coefficient (ICC) of absolute agreement based on a two-way mixed model [27]. An ICC over 0.70 indicated excellent test-retest reliability.

As joint impairment directly affects HRQoL, and as muskulo-skeletal assessments were available from the multidisciplinary visits, a Pearson’s correlation between the two HRQoL measures and the HJHS_2.1_ score was also assessed.

All analyses were performed using R software version 3.3.1. Continuous variables were presented as the means and standard deviations (SDs), which were compared between groups using Student’s t-test for independent variables, or as the medians with interquartile ranges, which were compared between groups using Wilcoxon rank-sum test according to the distribution normality. Categorical variables were presented as number of proportions, which were compared between groups using Pearson’s chi-squared test, Pearson’s chi-squared test with Yates continuity correction, or Fisher’s exact test, depending on the validity condition of each test.

## Results

### Linguistic adaptation findings

In January 2017, the French CHO-KLAT_2.0_ was reviewed by the Ivoirian team and judged to be understandable based on patient feedback. However, some difficulties were identified related to semantic, idiomatic (linguistic expression), and conceptual (disease-related) aspects of the CHO-KLAT_2.0_, and minor changes (mainly to prepositions) were made by the local team to improve understanding. This step was reviewed by both Belgian and Canadian teams, and after adjudication by the Canadian team and twin partners, 17 items were modified to improve the linguistic accuracy and comprehension for children and parents. An important issue was the terminology used to describe joint bleeding. The word “swelling” was chosen (“*gonflement* or *enflement*”), as this word is used in Côte d’Ivoire. The sentence “These things did not happen to you in the past 4 weeks” was adapted as follows to facilitate comprehension: “*Si ces choses ne sont pas arrivées dans ton cas, tu auras une autre réponse à choisir - If these things did not happen in your case, you will have another answer to pick*”. In addition, the example was adapted as follows: “*Je ne suis pas allé à l’école ces 4 dernières semaines et je n’ai donc pas écrit - I have not been to school in the past 4 weeks, so I have not responded*”. One item in the overall evaluation (the “summer programme” activity) was removed. The same modifications were applied to the self- and proxy-reported versions. Changes were made to 48.6% of the CHO-KLAT_2.0_ items in total based on the clinical expert review. The harmonization step was completed at the end of August 2017, resulting in the initial version of the child and parent Ivoirian CHO-KLAT_2.0_.

### Cognitive debriefing findings

Five children and parent pairs were interviewed. The mean age of the BWHs was 13.9 years (SD 1.6; range: 11.7–15.3). They all had HA, three with severe and two with moderate forms and two with familial and three with sporadic forms. One had an active inhibitor. All were treated on demand, and no home therapy was available. The participating caregivers included three mothers, one father, and one brother who was considered a parent proxy.

All CHO-KLAT_2.0_ items were globally well understood by the five boys and their parents. However, similar to the experience of the adaptation of CHO-KLAT_2.0_ in Brazil [28], we observed significant variability in reading skills among BWHs during the cognitive debriefing interviews. In the team review meeting after the 3rd and 5th cognitive debriefing interviews, consistent concerns were voiced, which resulted in four minor modifications to the wording of five items (14.2% of the items modified). The same changes were applied to the self-reported and proxy-reported versions. The mean CHO-KLAT_2.0_ score was 56.7 (SD 6.1) for BWHs and 47.7 (SD 15.6) for parents. The Ivoirian CHO-KLAT_2.0_ was finalized in September 2017.

### Validation results

**Fig. 1:**
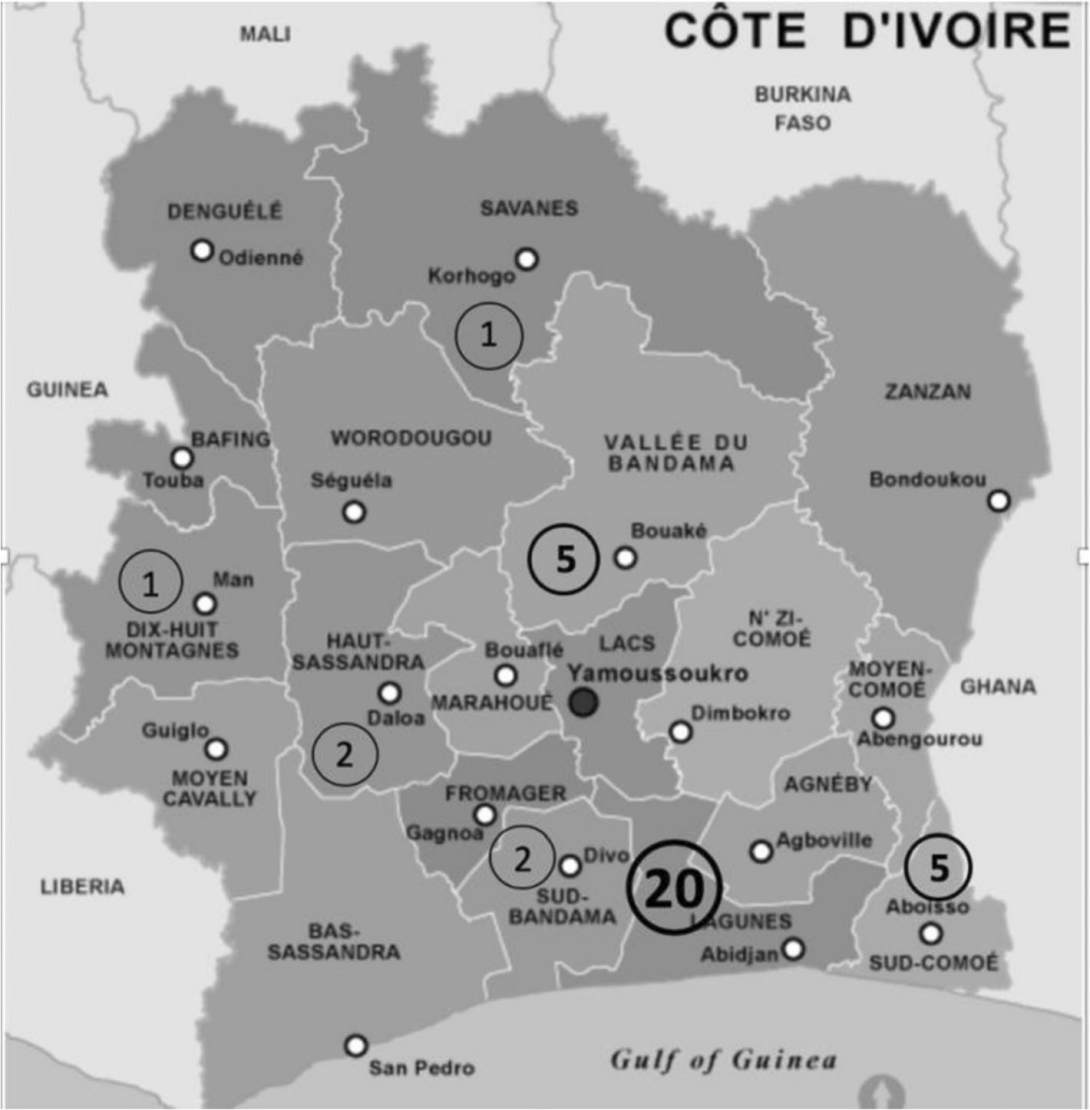
Geographical distribution of the Ivory Coast study subjects

The validity assessment of the final version of the Ivoirian CHO-KLAT_2.0_ included 37 BWHs. The clinical characteristics of the validity assessment sample are shown in Table 1. The mean age was 11.4 years (range 4.7–17.5 years). Joint impairment was observed in 84.8% of BWHs, and significant joint disease (HJHS > 10) was found in 66.7%. A majority (54%) of BWHs were living in the district of Abidjan where the Yopougon HTC is located. The geographical distribution of the study subjects is detailed in Fig. 1.

**Table 1:** Characteristics of the participating population for the validation of the Ivoirian CHO-KLAT_2.0_

	Total (*N* = 37) Mean ± SD, median [P_**25**_; P_**75**_] or n (%)	Self-reported outcome (*n* = 20) Mean ± SD, median [P_**25**_; P_**75**_] or n (%)	Proxy-reported outcome (*n* = 17) Mean ± SD, median [P_**25**_; P_**75**_] or n (%)	*p*-value
Boy’s age, years	11.4 **±** 3.6	13.6 **±** 2.1	8.8 **±** 3.1	< 0.001
Type of haemophilia				0.609
Haemophilia A	33 (89.2)	17 (85.0)	16 (94.1)	
Haemophilia B	4 (10.8)	3 (15.0)	1 (5.9)	
Clinical severity				0.234
Mild haemophilia	0 (0.0)	0 (0.0)	0 (0.0)	
Moderate haemophilia	3 (8.1)	3 (15.0)	0 (0.0)	
Severe haemophilia	34 (91.9)	17 (85.0)	17 (100.0)	
Inhibitor status				0.999
Previous inhibitor	1 (2.7)	1 (5.0)	0 (0.0)	
Current inhibitor	2 (5.4)	1 (5.0)	1 (5.9)	
Never had an inhibitor	34 (91.9)	18 (90.0)	16 (94.1)	
Boy’s age categories				< 0.001
4–7.9 years	8 (21.6)	0 (0.0)	8 (47.1)	
8–11.9 years	10 (27.0)	4 (20.0)	6 (35.3)	
12–17.9 years	19 (51.4)	16 (80.0)	3 (17.6)	
Proxy respondent for the boy				
Mother		/	12 (70.6)	
Father		/	5 (29.4)	
Familial haemophilia				0.157
Yes	22 (59.5)	14 (70.0)	8 (47.1)	
No (sporadic)	15 (40.5)	6 (30.0)	9 (52.9)	
BMI percentile	24.1 [9.1; 38.9]	28.5 [8.2; 46.2]	23.2 [10.4; 35.1]	0.479
Underweight (<5th percentile)	7 (18.9)	4 (20.0)	3 (17.6)	
Normal weight (5th–84th percentile)	30 (81.1)	16 (80.0)	14 (82.4)	
Proxy’s occupation				
Works at home			11 (64.7)	
Works outside home			6 (35.3)	
Proxy’s highest diploma				
Never went to school			2 (11.8)	
Primary school			13 (76.5)	
Secondary school			1 (5.9)	
Graduate school			1 (5.9)	
PedsQL total score	59.2 **±** 13.2	58.9 **±** 16	59.6 **±** 9.5	0.858
CHO-KLAT total score	52.3 **±** 9.4	51.3 **±** 9.2	53.5 **±** 9.8	0.491
HJHS total score^a^	16.0 [6.0; 23.0]	18.5 [11.8; 22.8]	15.0 [1.0; 23.5]	0.425

*BMI* Body mass index, *PedsQL* Pediatric Inventory of Quality of Life, *CHO-KLAT* Canadian Haemophilia Outcome-Kid’s Life Assessment Tool, *HJHS* Health Joint Hemophilia Score 2.1

^a^4 missing values (10.8%); *SD* standard deviation

All children attended school regularly, except one who left at age 10 due to severe arthropathy (this boy had the worst HJHS [41] of the cohort). One 16-year-old BWH was held back in primary school due cognitive impairment secondary to an intracranial bleed in infancy. To determine whether BWHs aged 8–12 years old were able to complete self-assessments, they were asked to read two simply worded short stories and to respond to three questions with multiple-choice answers to evaluate their reading and understanding.

All of the 15 BWHs aged 8–12 years were tested on their literacy prior to completing the self-reported questionnaire. Seven were able to complete the Ivoirian CHO-KLAT_2.0_ without assistance. Notably, 2 BWHs who were 13 and 14 years old experienced difficulties with the not applicable (NA) option (corresponding to “these things did not happen to you in the past 4 weeks”). We therefore proposed the inclusion of an example to illustrate and train boys how to answer the NA option so they could practice the use of the NA option. In total, 20 BWHs completed the self-reported questionnaire on their own; for BWHs who were under 8 years old, who had cognitive impairment or who failed the literacy test, their parents completed the parent proxy versions.

The 17 proxy respondents included 11 mothers and five fathers. One mother was the parent proxy for two sons with haemophilia. The details of the proxy participants are described in Table 1.

#### Distribution of the CHO-KLAT_2.0_ and PedsQL scores

BWHs reported a mean CHO-KLAT_2.0_ score of 51.3 (range: 35.5–71.7) and a mean PedsQL score of 58.9 (range: 37.0–92.4). The mean parent proxy score was 53.5 for the CHO-KLAT_2.0_ (range: 36.7–64.4) and 59.6 for the PedsQL (range: 40.3–75.0) (Table 1). No outliers were observed in the data.

#### Validity results

The correlation results between the Ivoirian CHO-KLAT_2.0_ and the PedsQL were expected to be similar to those in the original Canadian study (*r* = 0.59) [12] and are described in Table 2. The Pearson’s correlation between the child-reported Ivoirian CHO-KLAT_2.0_ and PedsQL scores at baseline revealed a moderate correlation. This finding confirms the validity of the final Ivoirian CHO-KLAT_2.0_ for self-reporting BWHs. However, in the proxy-reported data, we found no significant correlation between the Ivoirian CHO-KLAT_2.0_ and PedsQL scores. We identified which questions showed significantly different answers between the self-and proxy-reported questionnaires for both HRQoL measures. Differences were observed for answers regarding the emotional, social, and educational dimensions but not the physical health dimension.

**Table 2:** Pearson’s correlation between the CHO-KLAT_2.0_ and other instrument scores for the self-reported and proxy-reported outcomes (*N* = 37)

Instrument	Total (*N* = 37)		Self-reported outcome (*n* = 20)		Proxy-reported outcome (*n* = 17)	
	R	*p*-value	R	*p*-value	R	*p*-value
CHO-KLAT_2.0_ vs PedsQL	0.350	0.034	0.581	0.007	−0.050	0.850
CHO-KLAT_2.0_ vs HJHS	−0.392	0.024	−0.339	0.169	−0.414	0.125
PedsQL vs HJHS	−0.537	0.001	− 0.653	0.003	− 0.490	0.064
CHO-KLAT_2.0_ vs GQ1	−0.186	0.271	−0.367	0.111	−0.081	0.757
PedsQL vs GQ1	−0.341	0.039	−0.497	0.026	−0.164	0.529

*R* Pearson’s correlation coefficient, *CHO-KLAT*_*2.0*_ total score of the CHO-KLAT_2.0_ questionnaire, *PedsQL* total score of the PedsQL questionnaire, *HJHS* Hemophilia Joint Health Score 2.1, *GQ1* General question 1 of the CHO-KLAT_2.0_ questionnaire “How much are you bothered by your haemophilia?”

The degree of correlation between the HRQoL scores and the rating of the degree to which BWHs were bothered by their haemophilia was assessed (Table 2). We observed an inverse relationship between the child-reported Ivoirian CHO-KLAT_2.0_ scores and the global ratings of bother and between the child-reported PedsQL scores and the ratings of bother. No significant relationship was observed between the parent-reported scores and the global ratings of bother. The internal consistency of the Ivoirian CHO-KLAT_2.0_ items, as measured by Cronbach’s alpha measure, was 0.58 (95% CI: 0.42; 0.78) for the self-reported questionnaire and 0.65 (95% CI: 0.43;0.88) for the proxy-reported questionnaire.

#### Reliability results

The ICC between the T1 and T2 assessments was calculated for 19 self-reporting BWHs (one boy did not return the questionnaire at T2). The delay between T1 and T2 was an average of 8.16 days (range: 6–11 days, median: 8 days). The test-retest reliability was assessed in 17 proxy-parents. The proxy respondents answered the surveys with a mean of an 8.59 day interval (range: 6–13 days, median: 8 days). We observed an excellent ICC for both the self- and proxy-reported questionnaires. Detailed results are presented in Table 3.

**Table 3:** Reliability of the CHO-KLAT_2.0_ total score between the first and second assessment (*N* = 36)

	First assessment (T1) Mean ± SD	Second assessment (T2) Mean (SD)	ICC (95% CI)
Total (*n* = 36)	52.8 **±** 9.1	50.8 **±** 9.0	0.877 (0.734; 0.941)*
Self-reported outcome (*n* = 19)	52.2 **±** 8.7	50.1 **±** 8.8	0.879 (0.673; 0.954)*
Proxy-reported outcome (*n* = 17)	53.5 **±** 9.8	51.6 **±** 9.4	0.880 (0.694; 0.955)*

*ICC* intraclass correlation coefficient, *95% CI* 95% confidence interval

*p-value < 0.05 to test whether the ICC was > 0.70

One of 37 patients was excluded due to not completing the second assessment

#### Relationship between HRQoL and the HJHS

The correlation between the HRQoL measures and the HJHS_2.1_ score was assessed. The joint damage evaluation presented a weak inverse correlation with the Ivoirian CHO-KLAT_2.0_ score (Pearson’s *r* = − 0.339; *P* = 0.169) and a strong inverse correlation with the PedsQL score (*r* = − 0.653 and *P* = 0.003) among the self-reporting BWHs. For the parents, the Pearson’s correlations showed a weak correlation between the HJHS and the CHO-KLAT_2.0_ score (*r* = − 0.329; *P* = 0.230) and a very weak correlation between the HJHS and the PedsQL score (*r* = − 0.178; *P* = 0.494).

#### Participants’ experience with the CHO-KLAT_2.0_

BWHs gave the test an average rating of 8.9, while the proxy respondents gave the test an average rating of 9.4 (on a scale from 0 [very bad] to 10 [very good]). The average time required to independently complete both the CHO-KLAT_2.0_ and PedsQL surveys was 25.6 min for BWHs and 27.3 min for proxy respondents. Most participants (75.6%) did not perceive any items to be missing. One parent reported the need to tailor the questionnaire to different ages, another suggested adding the answer “I have no idea of the thoughts of the child”, and another suggested adding an item about the financial considerations of haemophilia. Two parents commented on their motivation to learn how to infuse clotting factor concentrates. The open-ended questions highlighted the haemophilia consequences that most affected the boys and their parents. Swelling (joint and muscle bleedings) and limitations in sports were the most frequent concerns reported. Only 1 BWH complained about the burden of infusions.

## Discussion

This study was conducted to develop a cultural and linguistic adaptation of the CHO-KLAT_2.0_ and test its validity and reliability in Côte d’Ivoire. The cultural and linguistic adaptation and validation processes required few resources and they are thus accessible for countries with financial restrictions.

Although the sample size was small, most (86%) of the BWHs diagnosed and monitored in 2018 in Côte d’Ivoire were included in the study. With 46% of the participants not living in the Abidjan area, we had a good representation of Ivoirian regions.

Unsurprisingly, the mean child-reported CHO-KLAT_2.0_ (51.3) and PedsQL (58.9) scores among the Ivoirian BWHs were lower than those observed in Canada (CHO-KLAT_2.0_ = 75.4 and PedsQL = 80.9) [13] and European countries (CHO-KLAT_2.0_ = 77.0 and PedsQL = 83.8) [14], where there is regular access to prophylaxis. The same results were found for the proxy respondents. The mean HJHS_2.1_ score of the 33 Ivoirian BWHs was clearly higher (indicating more joint damage) than those observed among BWHs from Canada [29]. Similar findings were recently described in a study comparing the burden of haemophilia in children from Canada with that of PWH in Brazil [29]. This finding underlines the gap in HRQoL between BWHs living in countries with economic resource limitations and those without, as well as the urgent need to improve prophylaxis access.

The final Ivoirian CHO-KLAT_2.0_ was moderately correlated with the PedsQL score in the child reports (Pearson’s *r* = 0.581), which is similar to the results of the Canadian study (*r* = 0.59) [12] and other cross-cultural validations of the CHO-KLAT_2.0_ (e.g.*,* in Brazil (*r* = 0.47) [16] and China (*r* = 0.67) [18]). This confirms the validity of the final Ivoirian CHO-KLAT_2.0_ among self-reporting BWHs. We found an inverse correlation between the degree to which BWHs were bothered by their haemophilia, the child-reported Ivoirian CHO-KLAT_2.0_ scores, and the PedsQL scores. The reason we found a stronger correlation with the generic measure instead of the specific measure (more relevant to haemophilia issues) could be due to the small sample size.

In the proxy reports, we found no correlation between the Ivoirian CHO-KLAT_2.0_ and PedsQL scores, nor was any significant relationship observed between the parent-reported scores and the global ratings of bother. Discordances were observed between the self- and proxy-reported responses for both HRQoL measures concerning emotional, social, and educational aspects but not physical health aspects. As there were no obvious differences (in terms of disease severity and treatment) between the self- and proxy-assessed BWHs, we hypothesized that the perception or understanding of the Ivoirian CHO-KLAT_2.0_ might have been problematic for some proxy respondents, potentially due to parents having greater difficulty estimating the social and emotional impact of haemophilia compared to the physical consequences of haemophilia for their children. This difficulty is well illustrated by one parent’s suggestion to add an answer option for “I have no idea of the thoughts of the child”. This conceptual barrier could be related to the local culture or the parents’ education levels. We carefully selected the children using a literacy test, yet we did not anticipate the possibility that some parents might also face literacy difficulties. As previously reported during the adaptation of the CHO-KLAT_2.0_ in Brazil [27], literacy assessment is very important considering the low number of BWHs who were able to complete the questionnaires without assistance. The age limit for children’s independent completion of the questionnaire remains to be determined in Côte d’Ivoire in view of some difficulties observed even among older BWHs in answering the questions. Assessment of the literacy of proxy respondents should also be considered prior to the administration of HRQoL questionnaires, and if required, assistance should be provided to parents to ensure their understanding and determine their perceptions of the questions. Further work is thus needed to develop and validate the appropriate literacy assessment tools for children and parents before the administration of the CHO-KLAT_2.0_ in Côte d’Ivoire. The test-retest reliability of the Ivoirian CHO-KLAT_2.0_ was high in both BWHs and parents, and the Ivoirian CHO-KLAT_2.0_ demonstrated good concordance over time. Finally, we identified interesting similarities (limitations in sports) and differences (only one boy complained about the burden of clotting factor infusions) between the concerns reported by Ivoirian participants and those reported by participants from countries where prophylaxis is a standard of care [14].

## Conclusion

Accurately assessing the haemophilia burden in Côte d’Ivoire is crucial when advocating for improved access to safe factor concentrates. The need for HRQoL outcome measures became urgent as low-dose prophylaxis with extended half-life products was initiated in 17 young BWHs in January 2018. Now, a cross-cultural adapted and validated Ivoirian version of the CHO-KLAT_2.0_ is available, baseline measurements can be obtained, the impact of prophylaxis in Ivoirian BWHs can be assessed, and the outcome of further interventions in this population can be measured. Finally, the ability to assess HRQoL should also enable the Ivoirian HTC to participate in multisite international haemophilia clinical trials. However, our experience from this study highlights that some measures should be applied to ensure an optimal understanding of quality of life questionnaires that have been developed in culturally distinct countries and that could be used in clinical studies assessing different treatment regimens.

## Data Availability

Available data are presented in the manuscript. The final Ivoirian CHO-KLAT_2.0_ is not available due to copyright restrictions.

## Abbreviations

BWH: Boy with haemophilia
PWH: People with haemophilia
CHO-KLAT: Canadian Haemophilia Outcome-Kids’ Life Assessment tool
PedsQL: Pediatric Quality of Life Inventory
Haemophilia A: HA/Haemophilia B: HB
HRQoL: Health-related quality of life
HTC: Hemophilia Treatment Center
BMI: Body mass index
HJHS: Hemophilia Joint Health Score
ICC: Intraclass correlation coefficient
NA: Not applicable
